# Design and acceptance of Rheumates@Work, a combined internet-based and in person instruction model, an interactive, educational, and cognitive behavioral program for children with juvenile idiopathic arthritis

**DOI:** 10.1186/s12969-015-0029-5

**Published:** 2015-07-23

**Authors:** Wineke Armbrust, Joyce J. F. J. Bos, Jeannette Cappon, Marion A.J. J. van Rossum, Pieter J. J. Sauer, Nico Wulffraat, Veera K. van Wijnen, Otto T. H. M. Lelieveld

**Affiliations:** Department of Pediatric Rheumatology, University of Groningen, University Medical Center Groningen, Beatrix Children’s Hospital, Groningen, The Netherlands; University of Groningen, University Medical Center Groningen, Center for Rehabilitation, Groningen, The Netherlands; Amsterdam Center for Rheumatology and immunology Reade, location: Dr. Jan van Breemenstraat, Amsterdam, The Netherlands; University of Groningen, University Medical Center Groningen, Beatrix Children’s Hospital, Groningen, The Netherlands; Department of Paediatric Immunology, University Medical Centre Utrecht, Wilhelmina Children’s Hospital, Utrecht, The Netherlands

**Keywords:** Juvenile idiopathic arthritis, Cognitive behavioral program, Physical activity, Acceptance, Self-management

## Abstract

**Background:**

Juvenile idiopathic arthritis (JIA) is a chronic rheumatic disease. Patients suffer daily discomforts such as pain, fatigue, stiffness, and mood disturbances. Their exercise capacity is decreased to a variable degree and physical activity levels may be impaired. To prevent long-term cardiovascular risks associated with JIA and medication, it is important to encourage physical activity. To achieve this we developed Rheumates@Work (R@W), a combined internet-based and in person instruction model, an interactive, educational, and cognitive behavioral program. The aim of this study is twofold: to describe the theoretical background and design of R@W based on Pender’s Health Promotion Model, and to assess its acceptance.

**Methods:**

We enrolled 8 to 13-year-old JIA patients, from 3 outpatients clinics in The Netherlands, in R@W. Inclusion criteria were a low disease activity (VAS physician <20 mm), comprehension of the Dutch language and absence of relevant co-morbidity. We assessed acceptance by measuring the participants’ commitment to the program, the level of interaction on patient’s initiative (f.e. mails send by the patient), technical aspects and satisfaction. Commitment was defined as the percentage of participants that completed the assignments and how much encouragement the participants needed for this. Satisfaction was measured with an anonymous questionnaire concerning f.e. time investment and perceived benefits. Costs were monitored.

**Results:**

Of the 64 patients we enrolled, 23 boys and 41 girls, 93.8 % completed the program. Participant-initiated interaction was seen in 10.7 %, 24.7 % send a mail because of technical problems. Eighty-two percent of the participants and 99 % of the parents liked the program, and 85 % of the participants indicated that they had learnt something, or quite a lot. Development costs of the program were low.

**Conclusion:**

The HPM is suitable for a behavioral intervention program such as R@W. Acceptance and satisfaction of R@W were high and the costs of the program were low.

**Trial registration:**

Trial Number: ISRCTN92733069

**Electronic supplementary material:**

The online version of this article (doi:10.1186/s12969-015-0029-5) contains supplementary material, which is available to authorized users.

## Background

Juvenile idiopathic arthritis (JIA) is the most common chronic rheumatic disease of childhood, with a various disease course [1–4]. Due to the chronic nature of the disease, it is important that JIA patients learn to manage their health and learn to deal with the consequences and symptoms like stiffness, fatigue, sleep, and mood disturbances of JIA on a daily basis [5–12]. The exercise capacity of children with JIA is impaired [13–15] and, in comparison to healthy peers, they spend less time on physical activities [16, 17]. Why exercise capacity is decreased in these patients is not clearly understood. In part it may be explained by disease activity and severity, but other multifactorial causes have been suggested and need to be investigated [13–15]. Activity levels in adolescent patients do not correlate well with JIA disease activity, indicating a complex cause for the low activity levels [16].

Daily physical activity plays an important role in preventing chronic conditions, including diabetes, cardiovascular diseases, and obesity [18–21]. Evidence is available which suggests that suffering from JIA leads to cardiovascular disease risk factors and the risk of cardiovascular dysfunction [22, 23]. Inflammation is one of the causes of atherosclerosis. Moreover, obesity also occurs more frequently in patients with JIA [22–24]. Improving physical activity in these patients is, therefore, of the utmost importance.

In order to achieve this aim, we developed Rheumates@Work (R@W) (Trial Number SRCTN92733069), a combined internet-based and in person instruction model, an interactive, educational, and cognitive behavioral program to increase physical activity in 8 to 13-year-olds with JIA. R@W emphasized the importance of coping strategies, self-efficacy, and self-management. Results of a single center pilot of R@W published in 2010 showed that the intervention resulted in an improvement of PA in those patients with low PA levels. It was also able to improve endurance and it was safe, feasible, and had good adherence [25]. The pilot showed limitations as lack of power, selection bias, fair baseline levels of PA and a possible seasonal influence. For this reason a multicenter trial (MCT) was performed. The content, length and lay out of the internet application was improved using the experience of the staff and opinion of the participants of the pilot. The development of an intervention is challenging and background information for such a process is hard to find in the literature.

The first aim of this paper is to assess the acceptance of R@W in terms of commitment, level of interaction, technical aspects, costs, effort, satisfaction, educational content, and the perceived benefits of the program. The second aim is to describe the theoretical background and the design of R@W. Results of the intervention on PA will be described separately.

## Methods

### The acceptance of Rheumates@work

#### Patients

All JIA patients aged 8 to 13, from the following departments or hospitals, were invited to participate: the Departments of Pediatric Rheumatology at Beatrix Children’s Hospital (BCH) and Wilhelmina Children’s Hospital (WCH) of University Medical Center Groningen and University Medical Center Utrecht, respectively, and the Reade Center for Rehabilitation and Rheumatology in Amsterdam. Approval was obtained from each center’s medical ethics committee. Inclusion criteria were: diagnosed with JIA, good comprehension of the Dutch language, and access to a computer and to the internet. Patients with high disease activity, defined as > 20 mm on a 0–100 mm physician visual analog scale, were excluded. They could, however, still be included after six month if inactive disease was achieved. Patients with a physical disability caused by another chronic disease were excluded. Each January and September, from 2011 until 2013 a new group with a maximum of 15 patients started. To measure the effect of R@W on physical activity, the patients were randomly assigned to an intervention group and a waiting list group with an electronic function of SPSS version 22. The waiting list patients could participate in the R@W program after a six-month waiting period. R@W ran from September to January or from January to June. The outcome of R@W on physical activity is beyond the scope of this paper. In this paper we measured acceptance based on the participation of all the children in the program without taking into account the results of the randomization.

#### Acceptance

Acceptance was described as commitment to the program, technical aspects, level of interaction and satisfaction.

*Commitment* to the program was measured by the number of participants who had completed the weekly assignments on Monday. We counted the number of participants who had only completed the assignments and the number of participants who had completed both the assignments and the theory. We monitored whether the participants had done the assignments, be it completely or incompletely (fantasy answers were regarded as incomplete). The results were expressed in the average of the percentage calculated for each week of patients who had completed the assignment by Monday. We also monitored how long it took for patients with a backlog to catch up again. We noted the individual reasons for incurring a backlog. We counted the number of participants who needed an e-mail on Friday to encourage them to complete that week’s assignments. We also registered attendance at the group sessions and the reasons for absenteeism.

The *technical aspects* of the program we monitored by collecting the e-mails dealing with technical problems, such as log-in problems, crash of the site, etc.

The *level of interaction* we monitored by counting all the e-mails that were sent by the patients to initiate contact with the supervisor, and by registering participation in the chat sessions.

We calculated the *costs* by adding the cost of developing the program, the staff costs when conducting the program, and by monitoring the financial consequences for the participants for example traveling expenses, money needed for a babysitter for the sibs.

At the end of the 14-week period, we evaluated *satisfaction* with the program by asking the participants and their parents to separately fill out an anonymous questionnaire (Additional file 1). The questions addressed were: 1. time investment (answered on a three-point scale with a neutral option), 2. educational impact, and 3. had the participants and/or the parent liked the program (answered on a four-point scale without a neutral option). We also asked the participants and their parents to make suggestions for possible improvements to R@W. Parents were asked whether R@W had had an effect on their children. We also invited the patients to share with us any positive or negative comments about R@W that they might have.

A participation rate of 80 % was defined as representing good commitment to the program. This meant that a mean of 80 % of all participants should have completed each week’s assignment by Monday or should have caught up on their backlog within two weeks, 80 % or more should have been present at the group sessions, and 80 % off all the participants should have fulfilled both theory and assignments completely. Satisfaction with the program was achieved if more than 80 % of the participants and parents were positive about all the items in the questionnaire.

#### The theoretical background of R@W

**Fig. 1:**
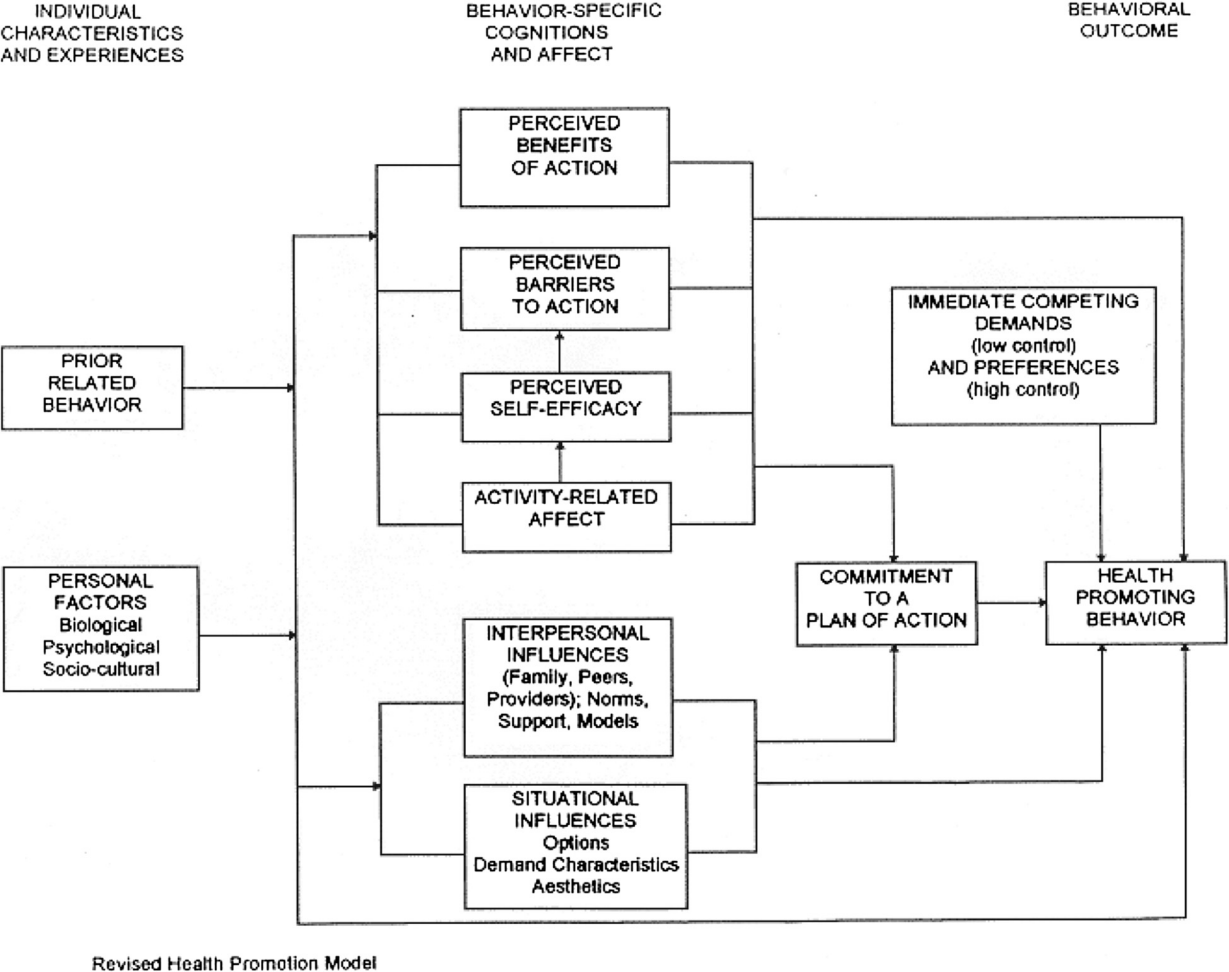
From: Pender, NJ, Murdaugh, C, Parsons, MA. (2011). Health promotion in nursing practice, 6th ed. Boston: Pearson, p. 45. With Permission by the authors

We based R@W on Pender’s Health Promotion Model (HPM) (Fig. 1) [26, 27]. This model explains the factors and relationships leading to the improvement of health and quality of life by means of health promoting behavior. The model stems from social cognitive theory and assumes that an individual’s ability to manage life events is based on the interaction between behavioral, interpersonal, and external factors [28]. Both the social cognitive theory and the HPM assume that people have the ability to shape their own future and to control the outcome. Therefore, the main concept of the HPM is self-efficacy. It refers to the strength of one’s faith in one’s own abilities, and one’s capabilities to complete assignments and achieve goals [29]. The HPM distinguishes three groups of determinants of health-related behavior: individual characteristics and experiences, behavior-specific cognitions and affect, and situational and interpersonal influences. Individual characteristics and experiences include age, gender, genetics, and prior behavior. These factors cannot be influenced. The behavior-specific cognitions and affect include perceived barriers, benefits, and self-efficacy. In the HPM these factors are the most important targets to influence health-related behavior. The situational and interpersonal influences are the social and environmental factors that also influence health-related behavior. The elements of the HPM, as shown in Fig. 1 and rendered in italics below, served as the basis for R@W. A plan of action, which is to be followed by the patient, is set up in order to achieve health-promoting behavior.

For a cognitive behavioral program to be successful it has to be adapted to patients’ experiences. In order to adapt the HPM to the specific requirements of JIA patients, we reviewed the burden of living with JIA from the patients’ point of view and integrated this with the contents of R@W. Two qualitative studies reported on the impact of living with JIA and what patients required to manage their disease [30, 31]. Although the target population in these qualitative studies did not represent the target population of R@W, the experience of the pilot study are in concordance with the literature (personal experience). JIA has a major negative physical impact. Patients frequently experience pain, fatigue, and disability. Reportedly, taking medicines is a problem because of the side effects. JIA patients sometimes encounter problems concerning their role in the family and their interaction with other children. To be able to increase their ability to manage the disease they feel the need for medical information and lifestyle management. They require strong social support and need to be actively involved in decisions concerning their own health.

#### The design of Rheumates@work

We designed R@W with a view to encourage physical activity, to stimulate self-management strategies, to improve self-efficacy, and to educate patients about JIA. For this purpose we applied the three groups of determinants of health-related behavior of the HPM, i.e. individual characteristics and experiences, behavior-specific cognitions and affect, and situational and interpersonal influences to the circumstances of children living with JIA. These patients need to overcome all barriers hindering health-promoting behavior and need to become aware of the benefits of such changes in behavior [26, 27].

The aim of the HPM is to improve health by achieving health-promoting behavior*.* In R@W we described *health-promoting behavior* in terms of primary and secondary outcome measures. We defined the primary outcome measure as improvement in the level of physical activity. We used the secondary outcome measure to improve the patients’ knowledge about JIA and to stimulate self-efficacy in dealing with different aspects of JIA, such as pain, setbacks, energy management, and taking medication. The starting point of the program was based on each patient’s *prior related behavior*, defined as the patient’s activity level as measured by a seven-day activity diary, and on *personal factors* defined in terms of gender, age, disease activity, joint damage, and functional disability. We assisted the participants in setting attainable goals to improve their health-promoting behavior. These goals were based on the individual patient’s activity level and personal situation.

In order to achieve health-promoting behavior, R@W addressed *behavioral cognitions and affect,* and *situational and interpersonal relations*. These we supplemented with topics such as pain, energy management, medication, and peer support, since we knew that these are important issues for patients in managing their disease [30, 31]. The different topics were: 1) Health education: With a view to improving disease management we taught the patients the fundamentals of JIA and the consequences of the disease for daily life. 2) Emotions and affect: We instructed the patients how to deal with the emotions and affect of having JIA. 3) Barriers to and benefits of being physically active: For example, we identified fatigue as a barrier to being active. Being active was turned into a benefit because physical activity leads to increased fitness, which in turn results in a decreased sense of fatigue. 4) Self-efficacy and perceived effect of physical activity: We instructed the patients about energy management, fatigue, accepting responsibility for taking medication, and dealing with pain. 5) Peer support: We stimulated the patients to use or to make more use of the support offered by family, friends, and school in order to become more physically active, and to enhance support concerning JIA in general. 6) ‘Smart’ goals were set: We taught the patients how to define and fine-tune specific attainable, realistic, and timely goals which varied with disease activity. 7) Setbacks: We taught the patients how to deal with setbacks in case of a relapse. 8) ‘Keep it up’: We coached patients to persevere with a view to future benefits.

We realized *commitment to a plan of action* by inviting the patients to sign a ‘declaration of commitment’ at the end of the first group session. During the last session we rewarded the participants by giving them each a Certificate of Participation.

R@W lasted 14 weeks. Each week we introduced a new topic through film, animation, puzzles, a spoken text, brain twisters, and/or assignments (see Table 1). Screenshots of the program are shown in Additional file 2. The choice of following the program alone or doing it together with their parents was up to the participants themselves to decide. R@W included four group sessions at which we addressed a different theme each time (Table 2). The maximum number of participants at a group session was 15 and the parents were expected to participate. All group sessions except for the third started with an introduction in which patients and parents were instructed together. After a short break the patients and parent were divided in separate groups. In the third session patients and parents were mixed and also friends and sibs were welcome. One chat session was organized during the 14 weeks. For this session one of the staff members acted as Buddy. Buddy discussed subjects like medication, feeling misunderstood, how it feels like to live with JIA etc.

**Table 1:** The outline of R@W; Pre-testing, the weekly themes and assignments and the after-testing

Week	Pre-testing	
2 to 4 before start	Rheumatologic evaluation	
	Fittest	
	Measuring PA by filling in an activity diary and wearing an accelerometer	
Week	Theme	Assignment
0	Start; Buddy, the cartoon figure who leads the child through the program, is introduced.	No Assignment
1	What is JIA and what is wrong with the immune system? The child is educated about the immune system by means of animations. How it works in normal situations, and in case of an auto-immune disease.	A skeleton called Hein, with a magnifying glass. When the child touches one of Hein’s joints on the screen, an X-ray image of that particular joint appears.
2	How to tackle disease-related participation problems. The schedules *problematic situation, thoughts, feelings, and action* are introduced. The child learns to cope with set-backs.	The child has to imagine a difficult situation related to the disease. Stepwise, he/she has to devise a positive solution.
3	Energy and condition. The child learns to cope with fatigue and learns to manage his or her energy level during the day and throughout the week.	The child makes a puzzle. He or she has to assemble a skeleton. After completing the skeleton Buddy pays the child a compliment.
		The child has to indicate how much energy he or she has by pointing to a color in a battery: red is empty, yellow is half full, and green is full.
		The child learns about the mechanism of exercising. While resting on the couch he or she has to count his or her heart rate and breathing frequency. Then he or she has to do the same after running around the house.
4	How to be active in a healthy way. The child learns to manage activities and to be active, and to stay active during times of active disease and during remission.	The child fills out his or her preferred activity, then he or she must indicate how he or she could improve his or her level of activity. For example, by adding an activity or by increasing the frequency.
5	Pain. How to differentiate between JIA and pain any child could experience, like muscle ache	The child has to describe situations in which he or she feels either cross, tired or sad. or happy. Then, he or she has to complete the following sentence: “When I’m cross, tired or I think of… I feel ..and I act ..”. Subsequently he/she has to change the cross/sad feeling into a positive feeling or action like calling a friend. The same goes for the situation in which the patient might feel pain.
6	Setting goals. The child looks at his or her own goal that was set in the first group session and is helped to formulate two SMARTs, i.e. specific, measurable, acceptable, realistic, and time- limited goals.	The child formulates two SMART goals to facilitate the goal that was set at the beginning. He or she has to answer the question: What do I want to achieve? How can I reach this goal? How often and how much time do I spend working on it? How much help do I need from other people? Will I succeed?
7	How to increase motivation by rewarding yourself. The child learns that when you achieve a goal it is good to reward yourself.	The goal is stabilized. The child has to answer the same set of questions as in Week 6. Next, the child thinks about how he or she could reward himself or herself after reaching his or her goal.
8	Taking responsibility. Barriers and benefits. The child learns about the barriers and benefits one meets when wanting to change activity-related behavior. The child is made aware of the benefits of being active.	The child has to click on an excuse machine, which gives excuses for not being active. After that the child has to type his or her best excuse and then he or she has to give the advantage and disadvantage of making this excuse.
		The next issue is physical limitations. The child has to hold his or her breath. Buddy also holds his breath and also becomes red in the face. This is a physical limitation because after several minutes the child must breathe out. Then the child has to provide two examples where h or she reached a physical limit. The child is taught that in some situations he or she can stretch his or her limit and that in some situations it is better to respect one’s physical limitations.
9	Activities and chatting. Every child has to fill out an activity diary for one day. All the children will then join in a chat session led by a supervisor to discuss their experiences.	The child has to fill out the activity diary for one day.
10	Doing things together and asking for help. Being active together with friends is more fun. The child learns what he or she can do with friends. And the child is stimulated to ask for help when it is difficult to do something because of JIA.	In three difficult situations, which have to do with JIA, the child has to decide whether or not to ask for help. For example, he or she is in a supermarket and has to take a product down from a high shelf while his or her joints are hurting. The child is encouraged to ask for help.
11	Talking about JIA. Being open about JIA can be beneficial to the child.	The child has to fill out a step plan about a situation in which a friend wants to skate but his or her ankle is hurting. Step1: Stop and think. Look at Hein and mark where it hurts. 2 What is the problem? 3. Which plans do I come up with? 4. Which plan do I choose? 5. Does the plan work?
12	Setbacks. The child learns that JIA is a disease that can fluctuate. The child learns how to adjust his or her goal when the arthritis becomes active again.	The child has set himself or herself three goals. The first at the start of the program and the others in Week 6. In this week he or she has to adjust the goals in case JIA is active. For example, cycling for 15 min instead of 30 min. Or cycling on alternate days.
13	Motivation. The child learns that it is important to persevere/to ‘keep it up’. One can keep up by developing motivation. The child is made aware that motivation is like a reward: when you reach your goals you have achieved something worthwhile.	In this week the child is motivated to adjust his or her goal if it has been reached in order to improve his or her capabilities. The child has to supply a motivation to adjust the goal. This is combined with a reward
14	During the last week everything the child has learnt is summarized	No assignments.
Week	After-testing	
2 after finish	Rheumatologic evaluation	
	Fittest	
	Measuring PA by filling in an activity diary and wearing an accelerometer

**Table 2:** Themes of the group sessions, the timing and the staff needed to organize a group session

GS	Theme/Timing		Staff (number)
1	What is JIA and what goes wrong with the immune system? /At the start	­- Plenary: Children and parents are taught what JIA is and are given tips on how to be physically active despite certain limitations.	PT (2), Phys (1)
		­- Children: setting goals for the next 14 weeks on the basis of their fittest level of activity	
		­- Parents: introducing themselves to one another and exploring the burden of having a child with JIA.	
		­- The children sign the declaration of commitment	
		­- The children formulate a goal for the health provider	
2	Excuses/week 4 or 5	­- Limits to physical activity. Some children have to be encouraged to be physically active and others have to be taught to taper physical activity. In both cases excuses are made up, which are discussed during this group session.	PT (1) Phys (1) S(1)
		­- Children: perform a role play and create a goal for the health professionals.	Psych (1)
		­- Parents: discuss the burdens that were mentioned in Group Session 1 and are given tips by the health professionals on how to deal with these burdens.	
3	Doing things together / Week 10	­- Skill games are played with the children’s friends and/ or their siblings, and with the parents.	PT (1) S (2)
4	What have we learnt and how to persevere / Week 14	­- Plenary: repetition of the theory. Doing a quiz	PT (1) Phys(1)
		­- The health professionals have to perform the goal set by the children previously.	
		­- Hand out the Certificates of Participation and crack open a bottle of bubbly to celebrate the successful completion of R@W.	

GS = Group session; P = Physiotherapist, Phys = Physician; Psych = Psychologist; S = student, +/- desirable, but not indispensible

R@W was designed as an interactive program. Participants were encouraged to ask Buddy questions by e-mail. Buddy was a cartoon figure (Additional file 2) and the main character in the program. He led the participants through the program and explained the thematic material by means of sound effects and texts displayed on the screen. His appearance changed every week and he served as a role model for keeping fit. He supported the children as a real-life buddy would.

We stimulated the participants to talk to family and friends about their disease. Moreover, we encouraged them to give a talk about JIA in class and to tell their schoolmates about this disease. With a view to easy accessibility we developed R@W as an internet-based program.

**Fig. 2:**
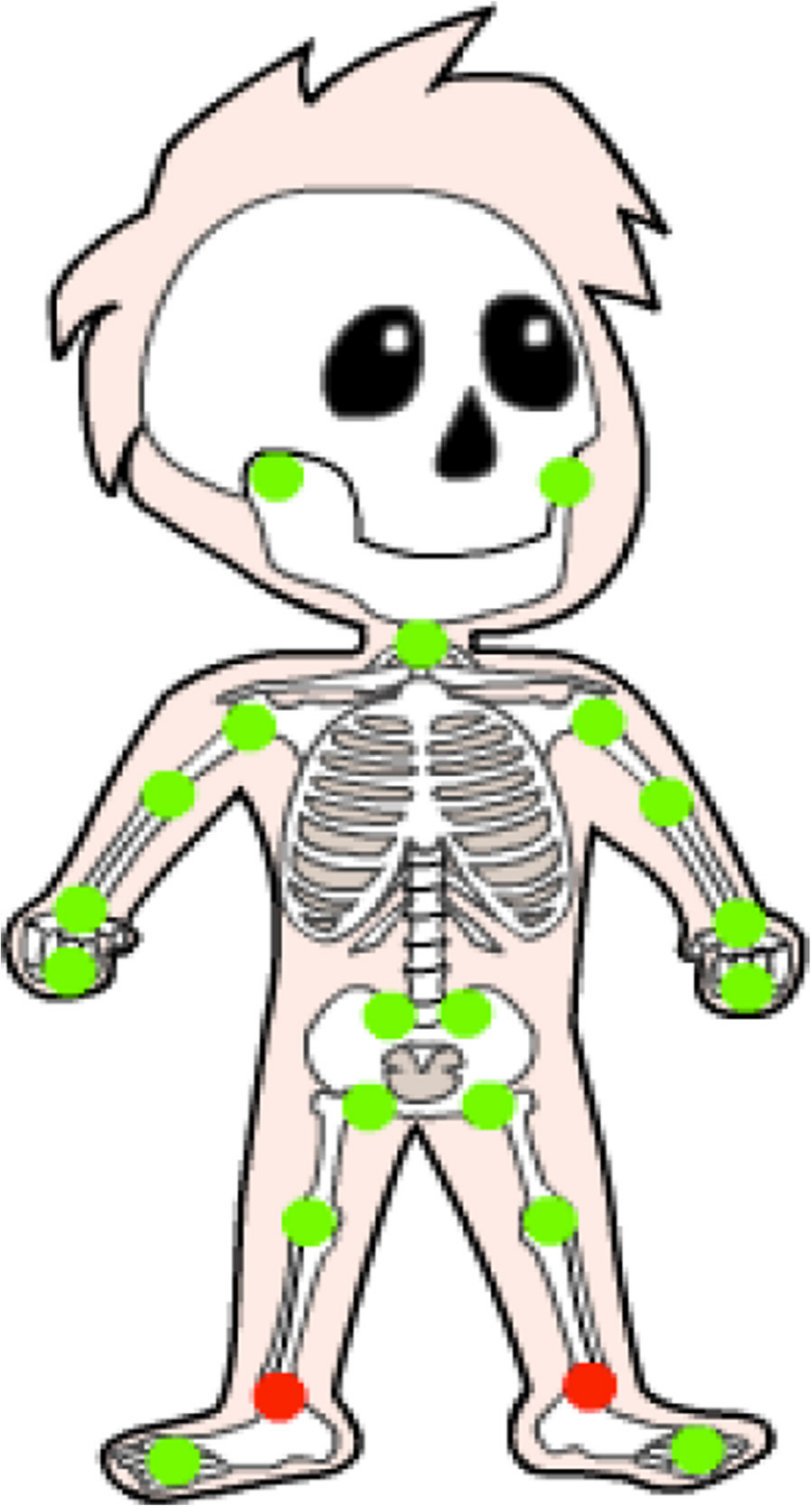
Skeleton “Hein”. The participant can see his/her joint status. Green means everything ok, orange means watch out for your limits and red means follow the instructions of the physician, your joint is damaged

R@W was personalized in the sense that we designed a personal page for each individual participant (Additional file 2). On it they could read the tip of the week, their personal goal, and the result of their preceding tests. At the beginning of the 14-week period, before actually starting the program, the patient’s joints were examined by the physician, who then colored them in on the patient’s personal page on a skeleton figure showing the most important joints (Fig. 2). In case of joint damage, the affected joint was colored red, a joint was colored orange in case of arthritis (in which case the patient was instructed to be aware of his or her limits), while in the absence of arthritis or joint damage, green was used (meaning no limitations). The color of the joint could change during the program on initiative of the physician, thus reflecting the current disease status.

Also prior to starting the program, the patient’s actual physical activity level (prior behavior) was measured with a seven-day activity diary and with an accelerometer. The results were correlated with the Dutch standard for physical activity [32]. This standard prescribes that every child should perform one hour of moderate to vigorous activity per day. Two of these hours should be aimed at improving the exercise capacity. The result of the activity diary was published on the patient’s personal page in terms of: “*You meet the Dutch standard for physical activity on x days a week”.* We assessed aerobic exercise capacity with the Bruce treadmill test (called fittest), which measures maximum endurance time [25, 33]. Results of the fittest were compared with reference values for Dutch children [34]. These results were displayed also added to the personal page of each participant as: ”Y*our physical fitness is excellent”*, or “Y*our physical fitness is moderate and requires improvement”*, or *“Your physical fitness requires considerable improvement”*. Based on the results of the patient’s level of activity and his or her physical fitness, and taking into account disease activity, we set a goal, together with the participant, for the next 14 weeks. Examples of goals were: *“I want to be just as fit as Buddy. To reach this goal I will ride my bike to school at least three days a week”.* Or *“To reach this goal l will take the dog on a brisk walk of at least 30 min every day.* After the goal was set, the patient signed the ‘declaration of commitment’ to the program.

The program started with the first group session. On the following Monday, the first internet week was released, and the web administrator released a new week on every Monday thereafter. A single click revealed the theory, the assignments, and each participant’s the tip of the week, and an e-mail was sent to draw attention to the fact that a new week was available on line (“*Hello Rheumates! A new week is now open and we will talk about …. Good luck!”*). On Wednesday, a standard reminder was sent to the participants by e-mail (“*Attention please! It’s Wednesday. Did you remember to do your assignments?”*). On Friday, only the patients who had not yet completed that week’s assignment received a reminder by e-mail (“*Hello! Beware, you’re lagging behind! You haven’t completed your assignment yet, and on Monday the next week will open. If you need help, just e-mail Buddy or call the R@W team”*).

## Results

**Fig. 3:**
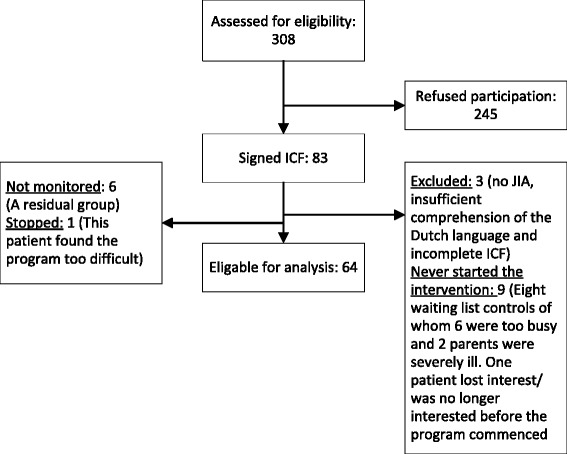
Flow diagram of the participants

We approached 308 patients by means of a letter of information about our study of whom 83 patients (27 %) were willing to participate (WCH 19 %, Reade 14 %, and BCH 88 %, respectively). We were not allowed by the Ethics Committee to ask patients and the parents the reason for not willing to participate in this study. Some patients and parents spontaneously indicated why they were not interested to participate. Reasons included: “*I am doing fine*”. “*My arthritis has been in remission for a long time*”. “*My health isn’t good at the moment*”. “*It’s too big a time investment*”. “*I’m already taking part in another study*”. Ultimately, we included 64 patients. Patient characteristics are presented in Table 3. We divided the participants into six groups spread out over two-and–a half years. Nineteen patients dropped out of the program for various reasons (Fig. 3), those were not included for analysis. For this study, only patients were included that participated in the intervention.

**Table 3:** Patient characteristics

	Participants
	N = 64
Man /woman	23 / 41
Age (years)	10.0 ± 1.4
Disease duration (years)	9.4 ± 1.4
Diagnosis (N)	
P-oligo	19
E-oligo	8
Poly Rf-	20
Poly Rf+	3
Psoriasis related	3
Enthesitis related	3
Systemic	8

P-oligo = persistent oligo-articular JIA, E-oligo = extended oligo articular JIA, Poly Rf- = Poly articular JIA Rheumatoid factor negative, Poly Rf + = Poly articular JIA Rheumatoid factor positive

### Acceptance

#### Commitment

By Monday, a mean of 53 participants (82.1 %) had fulfilled that week’s assignment completely and 8.6 (12.7 %) had caught up after two weeks (Table 4). A mean of 54.5 participants (83.7 %) per week had fulfilled the assignments completely by Monday and 8.3 (12.9 %) had handed in an incomplete assignment. Reasons for the backlogs were: away on holiday or school trip, illness, hospitalized, death of a family member, too busy, or no reason was given. At the end of the program, 60 participants (93.8 %) had completely fulfilled all the assignments for all 14 weeks on a proper way, also showing that they understood the meaning of the assignments.

**Table 4:** Results of acceptance of Rheumates@work; commitment and interaction

	Mean (SD)	%
Participants who had completed the weekly assignments by Monday	53.0 (6.5)	82.1 (10.4)
Participants who had completed the assignments by	54.5 (8.2)	83.7 (12.9)
Monday	8.3 (7.6)	12.9 (12)
-Completely		
-Incompletely		
Number of participants per week who sent an e-mail- of which type:	6.9 (4.6)	10.7 (7.2)
•Technical	1.7 (2.5)	
•Something not clearly understood	1.1 (1.1)	
•Friendly communication	2.6 (1.7)	
•Parents	0.5 (0.8)	
•Response to a reminder	1.1 (1.3)	
Present at the group sessions		
− 1 (reason for absence specified)	58 (3)	90.6 (4,7)
− 2(reason for absence specified)	57 (5)	89.1 (7,8)
− 3(reason for absence specified)	54 (7)	84.4 (10,9)
− 4(reason for absence specified)	47 (15)	73.4 (23,7)

On Friday, we send a reminder by e-mail to a mean of 17.9 participants (26.7 %). Group Sessions 1 to 3 were well-attended, i.e. by 54 to 58 (84.4 % to 90.6 %) of the participants. Group Session 4 was less well-attended by 47 patients (73.4 %). The reasons given were: on holiday, a sports tournament, a birthday party, influenza, no babysitter available to take care of the participant’s siblings, hospitalized, or weather conditions. In some cases no specific reason was given. There was no difference in the commitment between centers.

### Technical aspects

A mean of 1.7 (2.7 %) participants per week sent an e-mail concerning technical aspects. These included log-in details that had been forgotten, joints on the skeleton that had been colored incorrectly, or participants were unable to print a form which was needed for an assignment.

### Level of interaction

Every week a mean of 6.9 (10.7 %) participants took the initiative to send us an e-mail (Table 4). Of these messages 37.7 % were friendly e-mails communicating with Buddy. For instance: “*My goal is to run three times a week, but I find it so boring. But now I take my dog, and every time he has to pee I take a rest*”, “*I got a surfboard for my birthday, now my muscles are aching terribly, but I’m glad this pain isn’t due to the arthritis”* One boy kept on e-mailing long after he had completed the program “*Hi Buddy, I started a new school with many stairs, but I divided my energy so I got on very well”.*

Some participants sent e-mails because they were unclear about the assignments or about the dates of the group sessions. A couple of children reacted to the reminder e-mail by explaining why they had failed to complete that week’s assignment or they made a commitment about when they would complete the assignment.

Only 26.6 % of the participants took part in the chat sessions.

### Satisfaction

Overall, 81 % of the participants and 99 % of the parents liked R@W. Time investment was just fine or too short for 83 % of the participants. Seventy-one percent of the parents rarely, or never, needed to encourage their children. Ninety-five percent of the participants understood the theoretical topics well or very well. The level of the program and the assignments were perceived as adequate or too easy by 97 % to 89 % of the participants. Seventy-six percent of the parents reported that they had rarely or never needed to help their children. Eighty-five percent of the participants and 75 % of the parents indicated that they had learnt something, or quite a lot. The patients felt that they had learnt something about pain in 84 %, energy management in 84 %, arthritis in 87 %, and about setting goals in 85 % of the cases. Ninety-five percent of the parents felt that their child learnt something or quite a lot. For example: “*Coping with arthritis on a more conscious level, learning about energy management, limits, and physical activity”.*

Eighty-two percent of the participants liked R@W as did 99 % of the parents. Parents and participants wrote about positive aspects of R@W and gave suggestions to improve the program (Table 5). Group sessions and peer support was appreciated by 97 % of the parents and by 82 % of the children. There was no difference in the level of satisfaction between the three centers.

**Table 5:** Results of satisfaction; positive aspects and points for improvement

	Positive aspects of R@W	Points for improvement
Parents	- The children experienced that they were not the only ones with arthritis and that it helped to talk about it.	- Classification by age (8–10 and 10–12).
	- To talk about arthritis in a positive manner.	- More assignments for physical activities.
	- To have peer contact.	- More involvement of the parents during the program.
	- To receive education and information.	- Make the assignments less childish for the older kids and easier for the young ones.
	- To be understood by other parents and coaches.	- Create the possibility for the children to chat without the supervisors listening in.
	- To share experiences, and to receive tips.	- Fewer group sessions.
Patients	- I liked it very much.	- It was too childish.
	- I made a new friend.	- Buddy was not original.
	- I liked Buddy very much.	- I would like more physical assignments.

### Costs

A physiotherapist, a pediatric rheumatologist, and a psychologist developed the contents of the program with financial support amounting to € 10,000. Computer science students developed the technical part of the program as part of their Master’s degree by, for example, working on the application, the sound effects, cartoons, and the chat box. Students of the School for Web Design designed the lay-out of the program. A student teacher adapted the language used in R@W to suit children aged 8 to 13. The total direct costs amounted to less than € 1 500.

Staff deployment for the group sessions is described in Table 2. To manage the week’s progress, including the week opening, answering e-mails, and sending the reminder e-mails, required a time investment by one of the supervisors of 30 min per week.

The program consumed a maximum of one hour per week of the participants’ time. If possible, the group sessions were combined with a regular check-up visit to the physician.

## Discussion

JIA has a major impact on patients’ everyday life. Patients suffer from daily symptoms, which influence physical and psychosocial aspects of their lives. R@W is the first interactive cognitive behavioral program for children with JIA aimed at improving physical activity. Commitment to R@W was high and similar to the results of an internet-based self-management program for adolescents with JIA which supported participants by telephone [35] and higher compared to some other internet-delivered interventions for youth with health conditions [36]. The percentage of children that fulfilled the complete internet application in this multicenter study (MCT) was higher (93.8 %) compared to that of the pilot (82 %). Reason for this can be that the total duration of the MCT was shorter that the duration of the pilot namely 14 versus 17 weeks. Another reason can be that in the MCT the children were stimulated on a more tight and structural way to fulfill the assignments. The participation of the group sessions in the MCT was similar to that in the pilot [25].

- A reason for the high rate of commitment could be that the participants in our study were well-motivated and actively sought help in dealing with their disease. Only 30 % of the patients we approached were willing to participate. This could indicate that the participants represented the most motivated children. We did not find a difference in commitment between the three centers, even though the number of patients from each differed greatly. We were not allowed by the Ethics committee to ask why patients were not motivated to participate, so we do not know the reasons for the differences in participation between the hospitals. A possible explanation could be that the children in BCH knew the project leaders and thus were inclined to feel more committed. Another reason could be that in WCH many of the patients were already participating in one or more studies. Finally, a factor that could have influenced the participation rate at Reade was that many of the patients had just completed a rehabilitation program, which had been offered to many children diagnosed with JIA. In the BCH, where we had a participation of 88 %, almost all children completed the program. Potentially less motivated children also completed the program.
- We based the topics on what we knew from the literature as being the burden of living with JIA. R@W reflects the needs of the patients [30]. This probably leads to high commitment.
- The high commitment could also be explained by the declaration of commitment we asked the participant to sign. Possibly this made them adhere to the program more than they might have done otherwise. According to the HPM it is important to literally commit oneself to the program [26].
- R@W lasted 14 weeks with a time investment of at most one hour a week, which seemed reasonable to the participants. Although the role of duration and time investment in participants’ adherence is not explicitly described in the literature, it could be one of the factors influencing commitment [36].
- Another reason might be the interactive character of the program, which could have had an engaging effect on the participants. The value of interaction for the strength of patient commitment has not been investigated in the literature. Our analysis showed that more than a third of the participants had some kind of contact with the supervisors. In most cases this contact centered on reminder e-mails. We did not investigate whether this led to increased commitment or not.
- We point out that our program was based on an existing theory developed for the express purpose of changing health-promoting behavior.
- Finally, R@W was combined with group sessions, which involved a limited number of participants at a time. This could have increased participants’ commitment and adherence to the program as well as that of their parents. In a study with adolescent patients, parents were less compliant than their adolescent children. In our study, however, we found that parents of our patients aged 8–13 years, were well-motivated to complete the program [35]. The parents of children in the age of 8 to 13 may feel more responsible for the education of their children than parents of adolescents. In our study, parents liked the program and appreciated the peer support. In the case of R@W, 95 % of the parents felt that their child had learnt something, which could be a motivation for commitment.

Attendance of the group sessions was also good, but less compared to the internet program itself. An explanation could be the time investment to come to the hospital - some patients had to travel as much as one and a half hours to reach the hospital. Group sessions were organized on the participants’ school-free afternoons, which obliged them to choose between sports, birthday parties, or some such activity, and the group sessions. Participants could complete the internet assignments in their own time. Moreover, not all parents were able to take time off work or to combine the group sessions with the obligations for siblings.

As a result of the high satisfaction rate of the participants and their parents, we conclude that the contents of R@W met their needs for education and skills to deal with the program. Most of the suggestions given to improve the program related to the age categories. The age range of 8 to 13 seemed to be too wide and dividing the group in 8 to 11 and 11 to 13 could be a better option for the program. The theory and assignments appeared to be a bit difficult for the youngest and too childish for the oldest participants. If implementing R@W as a standard educational tool for all patients with JIA, we would suggest either selecting patients aged 9 to 11 years, or developing two different programs – one for 8 to 11-year-olds and another for 11 to 13-year-olds. Although patients and parent were positive on the effect of the program the effect on PA, effects on health related quality of life and fatigue need to be evaluated.

The costs of the program were relatively low. The time investment for both staff and participants seemed reasonable. Both the patients and their parents were satisfied.

In our opinion, the HPM is a suitable model to design a behavioral intervention program such as R@W. Acceptance of the program was high and the costs and effort of the staff to manage it, once the program had been developed, were low.

This study does, however, have its limitations. We did not study the reasons why patients were unwilling to participate and, therefore, we cannot assess whether or not our results were influenced by certain biases. Another limitation is that we did not measure knowledge before and after the program was completed, so that we were unable to check the increase in knowledge reported by the participants and their parents. Social desirability could be a limitation of the results of the measurement of satisfaction. Although the participants were asked to be critical on the program, for reason that the feedback should be used for further improvement of the program, we cannot exclude that the participants gave social desirable answers. A final limitation could be that only patients with low disease activity were included. This was done because we aimed with R@W to improve physical activity. Children with high disease activity at the start of the program will improve during 14 weeks on their PA levels simply by reduction of disease activity. Selection of children with low disease activity could have been a bias for the result of acceptance.

## Conclusions

We conclude that R@W, an interactive combined internet-based and in person instruction model, cognitive behavioral program, stemming from a social cognitive theory such as Pender’s HPM, is a way to educate children, to stimulate them to be more active, teach them self-management skills, and to provide them with peer support. Commitment and satisfaction on the part of the participants were high and the costs were low.

## Consent

Written informed consent was obtained from the patient’s guardian/ parent/next of kin for the publication of this report and any accompanying images.

## Additional files

Additional file 1:
**Questionnaire that was filled in by the patients after completing the program.** (DOCX 30 kb) Additional file 2:
**Screenshots of the program.** (DOCX 341 kb)
