# Supplementary material for: Design and acceptance of Rheumates@Work, a combined internet-based and in person instruction model, an interactive, educational, and cognitive behavioral program for children with juvenile idiopathic arthritis
Source: Pediatr Rheumatol Online J. 2015 Jul 23;13:31. doi: 10.1186/s12969-015-0029-5 (PMC4511536; doi:10.1186/s12969-015-0029-5)
Supplement: Additional file 1: — Questionnaire that was filled in by the patients after completing the program. (DOCX 30 kb) [file 12969_2015_29_MOESM1_ESM.docx]

**Appendix 1: Questionnaire that was filled in by the patients after completing the program**

**Questions about the pre testing:**

1. Filling in the number questionnaires during the pre-test was
   Very boring – boring – nice – very nice
2. I understood the questionnaires
   Very bad – bad – good – very good
3. The fittest was
   Very annoying – annoying – great – very great
4. Wearing an actical was
   Very annoying – annoying – great – very great
5. Rheumatologic check-up was
   Very annoying – annoying – great – very great

**Questions about the internet application**:

1. Did the program fulfill your expectations?
   Totally not – not – a little – I did not have expectations
2. The level of the program was?
   To hard – just fine – to easy
3. I think of Buddy as
   Very stupid – stupid – nice- very nice
4. I understood the subjects of R@W
   Not at all – not – good – very good
5. How did you judge the time investment?
   To much – just fine – not enough
6. The assignments were
   To hard – just fine – to easy
7. I was able to make the assignments alone
   Never – sometimes – often – always
8. Did you often think of your goals during the course?
   Never – sometimes – often – always
9. Did you need the reminder on Wednesday to finish the assignments?
   Never – sometimes – often – always
10. I learnt from the assignments
    nothing – very little – little – much
11. I learnt from the theory
    Nothing – very little – little – much
12. I learnt about pain
    Nothing – very little – little – much
13. I learnt about managing my energy
    Nothing – very little – little – much
14. I learnt about arthritis
    Nothing – very little – little – much
15. I learnt about setting goals
    Nothing – very little – little – much
16. Can you tell us what you learnt about pain, energy, arthritis and setting goals?

**Questions about the group sessions**

1. I learnt from the group sessions
   Nothing at all – nothing – much – very much
2. Do you think that the group sessions fit to the internet program?
   Not al all – not – much – very much
3. I liked group session 1
   Not al all – not – much – very much
4. I liked group session 2
   Not al all – not – much – very much
5. I liked group session 3
   Not al all – not – much – very much
6. I liked group session 4
   Not al all – not – much – very much
7. Which group session you liked the most and why?
8. The number of group sessions was
   To many – just fine – to less

**General questions:**

1. How do you like R@W?
   Don’t like it at all – don’t like it – like it – like it very much
2. Before I started with R@W I knew what I had to expect
   Not al all – a little – much – very much
3. The information flyer was
   Very unclear – unclear – clear – very clear
4. The information letter was
   Very unclear – unclear – clear – very clear

**Essay questions**

1. Who took the initiative to participate in R@W?
2. What did you learn from R@W?
3. What did you learn about pain?
4. What did you learn about talking about JIA?
5. What did you learn about energy?
6. What did you learn about setting goals?
7. What grade do you give R@W? 1 is very, very bad and 10 is perfect.
8. What else do you want to tell us?
