# Supplementary material for: Design and acceptance of Rheumates@Work, a combined internet-based and in person instruction model, an interactive, educational, and cognitive behavioral program for children with juvenile idiopathic arthritis
Source: Pediatr Rheumatol Online J. 2015 Jul 23;13:31. doi: 10.1186/s12969-015-0029-5 (PMC4511536; doi:10.1186/s12969-015-0029-5)
Supplement: Additional file 2: — Screenshots of the program. (DOCX 341 kb) [file 12969_2015_29_MOESM2_ESM.docx]

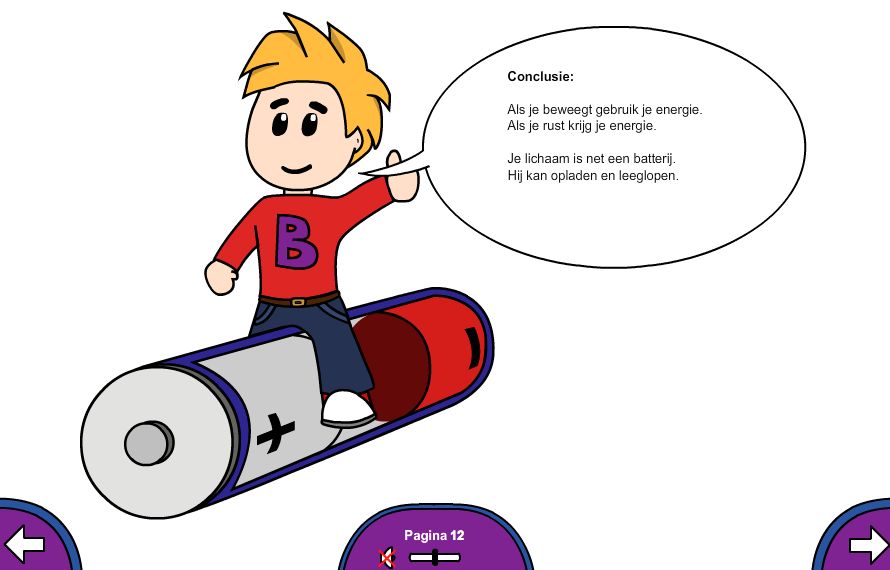


*Screenshot of the program; Buddy on a battery. He explains when you are active you use energy. By resting energy will grow. Your body is like a battery. It can charge and it can run down. When you are physical active on a regular base your battery will become bigger. You will have more energy*

.

*Example of a home page. The physicians opinion of the joins combined with the results of the fittest and the results of the activity level combined will lead to a goal for the child*


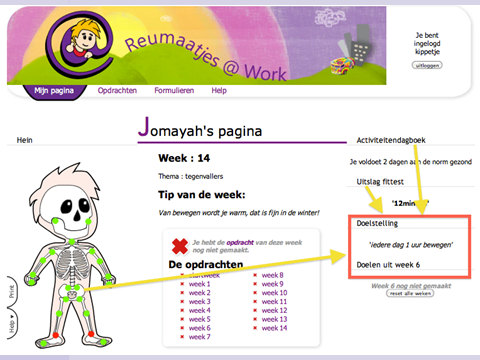


*.*


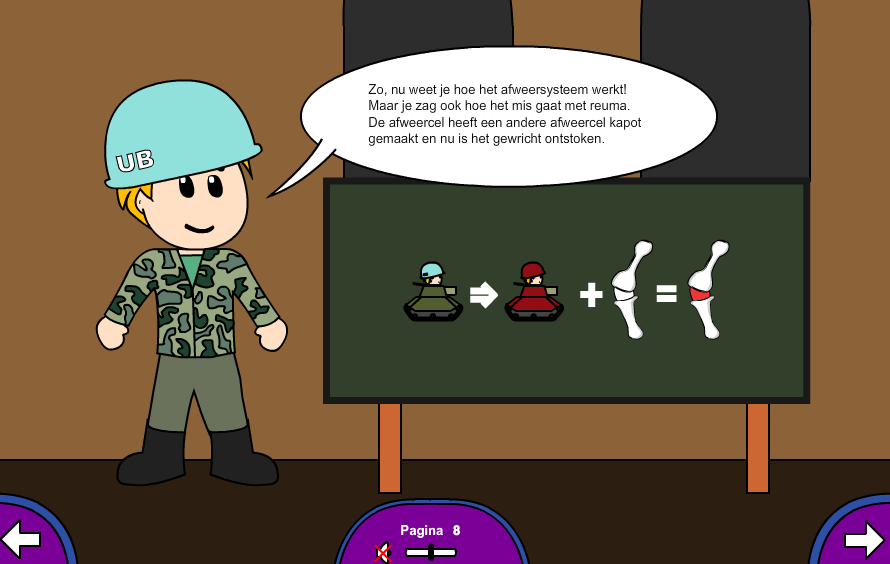


*Buddy is explaining the immune system. The body has soldiers to protect against microbes. Sometimes the good guys (green) become bad guys (red) and they will fight the body. This can lead to arthritis.*

*Appendix 2 Screenshots of the program*
